# Macrophages, Foreign Body Giant Cells and Their Response to Implantable Biomaterials

**DOI:** 10.3390/ma8095269

**Published:** 2015-08-28

**Authors:** Zeeshan Sheikh, Patricia J. Brooks, Oriyah Barzilay, Noah Fine, Michael Glogauer

**Affiliations:** Faculty of Dentistry, Matrix Dynamics Group, University of Toronto, 150 College Street, Toronto, ON M5S 3E2, Canada; E-Mails: patricia.brooks@utoronto.ca (P.J.B.); oriyah.barzilay@mail.utoronto.ca (O.B.); noah.fine@utoronto.ca (N.F.); Michael.Glogauer@utoronto.ca (M.G.)

**Keywords:** biomaterials, foreign body reaction, macrophages, implantable materials, cellular and tissue response, cell-material interaction

## Abstract

All biomaterials, when implanted *in vivo*, elicit cellular and tissue responses. These responses include the inflammatory and wound healing responses, foreign body reactions, and fibrous encapsulation of the implanted materials. Macrophages are myeloid immune cells that are tactically situated throughout the tissues, where they ingest and degrade dead cells and foreign materials in addition to orchestrating inflammatory processes. Macrophages and their fused morphologic variants, the multinucleated giant cells, which include the foreign body giant cells (FBGCs) are the dominant early responders to biomaterial implantation and remain at biomaterial-tissue interfaces for the lifetime of the device. An essential aspect of macrophage function in the body is to mediate degradation of bio-resorbable materials including bone through extracellular degradation and phagocytosis. Biomaterial surface properties play a crucial role in modulating the foreign body reaction in the first couple of weeks following implantation. The foreign body reaction may impact biocompatibility of implantation devices and may considerably impact short- and long-term success in tissue engineering and regenerative medicine, necessitating a clear understanding of the foreign body reaction to different implantation materials. The focus of this review article is on the interactions of macrophages and foreign body giant cells with biomaterial surfaces, and the physical, chemical and morphological characteristics of biomaterial surfaces that play a role in regulating the foreign body response. Events in the foreign body response include protein adsorption, adhesion of monocytes/macrophages, fusion to form FBGCs, and the consequent modification of the biomaterial surface. The effect of physico-chemical cues on macrophages is not well known and there is a complex interplay between biomaterial properties and those that result from interactions with the local environment. By having a better understanding of the role of macrophages in the tissue healing processes, especially in events that follow biomaterial implantation, we can design novel biomaterials-based tissue-engineered constructs that elicit a favorable immune response upon implantation and perform for their intended applications.

## 1. Introduction

**Figure 1:**
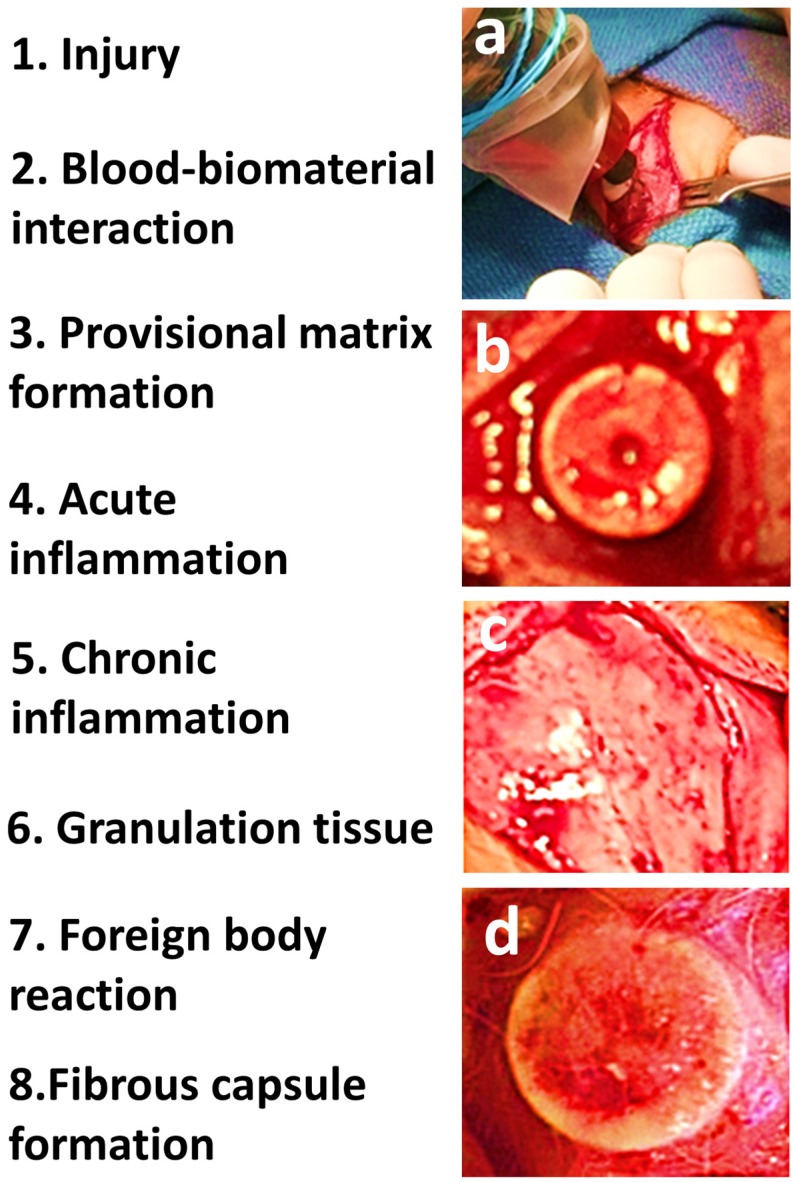
The sequence of host reactions upon implantation of biomaterial device *in vivo*. (**a**) Surgical incision and implant being placed causing injury; (**b**) blood-biomaterial interaction upon implantation; (**c**) inflamed soft tissue; (**d**) implant enclosed by fibrous capsule.

Biomaterials are engineered to take a form that can work alone or as part of a complex system providing direction to the course of any therapeutic procedure by regulating interactions with components of living systems [1,2]. Biomaterials are used to restore or augment the physiological function of diseased or damaged soft and hard tissues via replacement or regeneration [3,4,5,6]. Following the implantation of biomaterials *in vivo*, host reactions incorporate a combination of many processes including, blood-material interactions, provisional matrix formation, inflammation (acute then chronic), development of granulation tissue, foreign body reaction, and fibrous capsule development (Figure 1) [7,8,9,10,11]. 

Blood/biomaterial interactions begin to occur concurrently after bio-implantation, with protein adsorption to the biomaterial surface and the development of a blood-based transient provisional matrix (initial thrombus at the tissue/material interface) that forms on and around the biomaterial [12,13]. The provisional matrix is rich in cytokines, growth factors, and chemo-attractants that are capable of recruiting cells of the innate immune system to the injury site [14]. Following this provisional matrix formation, acute inflammation, and subsequently, chronic inflammation occur sequentially. The degree of these responses is dependent on the extent of injury during the implantation procedure [14]. The presence of neutrophils (polymorphonuclear leukocytes, PMNs) characterizes the acute inflammatory response. Degranulation of mast cells along with histamine release and fibrinogen adsorption mediates the acute inflammatory responses to implanted biomaterials [15,16]. Interleukin-4 and 13 (IL-4, IL-13) are released from the degranulating mast cells and play a role in determining the extent and degree of the subsequent development of the foreign body reaction [17,18]. Inflammatory responses to biomaterials may be modulated by histamine-associated phagocyte recruitment and adhesion to the implant surfaces facilitated by adsorbed fibrinogen [14,19,20].

Following acute inflammation, the recruitment of other inflammatory cell types to the implant site can lead to a chronic inflammatory state. Foreign body reactions, a type of chronic tissue inflammation, describe the presence of foreign body giant cells (FBGCs) at the biomaterial interface [10,14,21]. A biomaterial based acute inflammatory response usually resolves within less than one week [10]. With biocompatible implanted materials, early resolution of the acute and chronic inflammatory response occurs with the chronic inflammatory generally lasting no longer than two weeks and being confined to the implantation site. Persistence of the acute inflammatory response state beyond a three-week period usually indicates an infection [14]. After the resolution of acute and chronic inflammatory responses has occurred, granulation tissue is seen and confirmed by the presence of macrophages, fibroblast infiltration, and neovascularization in the new tissue. Granulation tissue may be a precursor to fibrous capsule formation and is separated from the implanted biomaterial device by the cellular components of the foreign body reaction (consisting of macrophages and FBGCs) [22]. There are many ways in which events can be altered to improve levels of tissue remodeling and reduce or eliminate fibrous tissue formation [23,24,25].

Multinucleated giant cells (MGCs) are a group of cells that are derived from the monocytic lineage, and are associated with bone loss, chronic inflammation, granulomatous disease and tumors [26]. Monocytes undergo fusion with one another in the presence of stimuli to form cell types that are present in both health and disease. Osteoclasts, bone-resorbing cells, are one of the better-characterized members of the MGCs. Osteoclasts work in tandem with osteoblasts, cells responsible for bone deposition, in the process of bone remodeling. The other member of the MGC class, the macrophage, is a prodigious phagocytic cell which responds to endogenous stimuli that are created after injury or infection. Macrophages were previously considered to be the first line of defense to infectious microorganisms, but more recently their roles in homeostasis and wound remodeling are being elucidated. This article focuses on the origin, role and response of macrophages to the implanted biomaterials and how this may affect their performance *in vivo*. The discussion presented will provide directions as to how these concepts can be integrated into biomaterial design to allow the creation of novel immuno-informed biomaterials that incorporate specific design principles to actively modulate the immune response to implanted biomaterials.

## 2. Origin and Role of Macrophages

**Figure 2:**
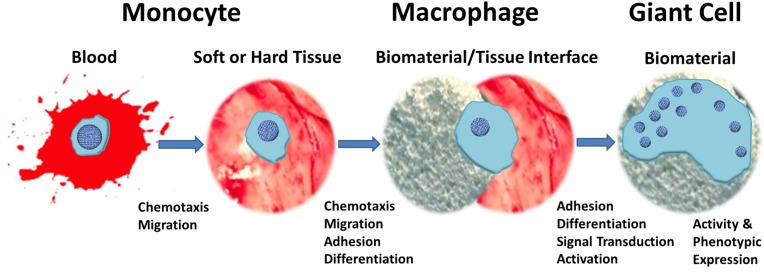
A schematic depiction of the transition of a blood-circulating monocyte to biomaterial/tissue interface-adherent monocyte/macrophage to foreign body giant cell.

Monocytes form from myeloid progenitor cells that give rise to monoblasts, pro-monocytes and finally monocytes. The induction of this cell differentiation occurs with the presence of colony-stimulating factors (CSFs) that are secreted by stromal cells in the blood and tissues [27]. Granulocyte-macrophage, granulocyte, and macrophage CSFs are thought to be involved in spreading, motility, and cytoskeletal reorganization [28]. Signaling by these cytokines allows these monocytes to leave the bone marrow and enter the bloodstream where they remain until being signaled to enter tissues by chemo-attractants (Figure 2). With respect to their phenotype and function, macrophages are very heterogeneous, a characteristic which is determined by signaling molecules and the cellular environment. Stimuli that produce an early macrophage response are typically from innate immune cells. Additionally, macrophages can respond to signals from cells that are antigen-specific [29]. Macrophages are also capable of producing factors that alter their own phenotype through autocrine signaling. Macrophages can be classified as classically activated, wound healing, and regulatory.

Classically activated macrophages, also called M1 macrophages, are produced during a cell-mediated immune response [30,31]. The presence of interferon-γ (INFγ) and tumor necrosis factor (TNF), or lipopolysaccharide (LPS) creates a macrophage population that secretes pro-inflammatory cytokines [32,33]. Natural killer cells produce INFγ, in response to infections in order to activate macrophages, causing them to secrete their own pro-inflammatory cytokines. This creates a group of macrophages with microbicidal and tumoricidal functions. T-helper (TH) cells are also able to provide a longer-lasting source of INFγ, where the T-cells act in an antigen-specific manner, but the macrophages that they activate can kill, irrespective of antigen specificity. Originally, classically activated macrophages were defined as requiring TNF, where a ligand of the toll-like receptor (TLR) acts to induce the transcription of TNF [34]. Some TLRs, however, have been reported to induce expression of both TNF and INF when activated. Classically activated macrophages play an important role in host defense through secretion of IL-12 and IL-23, high levels of inducible nitric oxide synthase and pro-inflammatory cytokines Il-1 β, IL-6, and TNFα [35,36,37]. However, this burden of inflammatory cytokines can also cause damage, with production of IL-1, IL-6, and IL-23 leading to an increased number of TH17 cells releasing IL-17. IL-17 release contributes to polymorphous leukocyte recruitment, which can lead to immunopathology, such as rheumatoid arthritis and inflammatory bowel disease.

Wound healing or reparative macrophages make up a group of cells which, like classically activated macrophages arise due to innate or adaptive signaling mechanisms. These cells were formerly called alternatively activated macrophages, or M2s. However, the cells are more associated with repair than defense [38,39]. Early on during tissue injury, IL-4 is released by mast cells and basophils, promoting the formation of macrophages that promote wound healing by creating components of the extracellular matrix [40,41]. These macrophages do not act as antigen presenting cells, produce very small amounts of pro-inflammatory cytokines, and are inefficient at producing radicals [42]. These macrophages express low levels of IL-12 and IL-23, with high levels of IL-10, an anti-inflammatory cytokine. The adaptive immune response can also produce IL-4, and is proposed by some to be the predominant pathway for the development of wound healing macrophages. TH2 cells release IL-4 and IL-13 to promote the formation of repairing macrophages. While wound repair is generally beneficial, when there is dysregulated activity by the wound repairing macrophages, tissue fibrosis may occur [43].

Regulatory macrophages occur as a result of either an innate or adaptive immune response. They appear to work to dampen the immune response and limit inflammation through anti-inflammatory cytokine IL-10 release [44]. The response of the body to stress, here considered part of the innate immune response, leads to the release of corticosteroids, which can work to inhibit pro-inflammatory cytokines [45]. Phagocytic abilities are intact in regulatory macrophages in the presence of glucocorticoids. Additionally, after phagocytosing apoptotic cells, TGFβ expression can lead to the immune regulatory activities of this cell type [46].

Macrophages display an interesting characteristic known as macrophage plasticity. Upon differentiation into one of the various macrophage subtypes, these cells, are not terminally differentiated, and can respond to local microenvironment signals [47,48,49,50]. Many different types of signals exist to promote phenotype switching, and vary from cytokines to the presence of a foreign body. Classically activated macrophages have been shown to become resistant to TLR responses, stop producing pro-inflammatory cytokines, and retain the ability to release anti-inflammatory IL-10 [50,51,52]. In the opposing situation, wound-healing macrophages are able to express the cytokine phenotype characteristic of classically activated macrophages after exposure to IFN and LPS. This complex state of macrophage plasticity likely represents a type of protective feature which allows for healing and repairs with the ability to form a rapid response to a pathogen. Additionally, it appears that this alteration of previously differentiated macrophages plays a role in the foreign body giant cell (FBGC) response.

## 3. Fusion Mechanism for the Formation of MGCs

Monocyte membrane fusion occurs in the formation of FBGCs, where adhesion proteins, membrane lipid rafts, and actin rearrangement are critical in the final step of membrane fusion [53]. Cell fusion at its structural level requires both the approximation and disruption of cell membranes in order for them to fuse. One modulator of fusion includes calcium ions that bind Soluble NSF Attachment protein REceptors (SNAREs). SNAREs are broken down into target membrane proteins (t-SNAREs) and secretory vesicle-associated proteins, (v-SNAREs). With calcium ions, the SNAREs interact and self-assemble into a ring conformation to form conducting channels. The calcium bridges the apposing bilayers, releasing water from hydrated Ca^2+^ ions which destabilizes the membrane in order for fusion to occur [54]. Fusion of cells is also proposed to occur due to the recruitment of membrane lipid rafts that possess the necessary adhesion molecules and align them with opposing membranes, using the actin wall as a supporting platform [55,56]. It has also been documented that fusion pores are generated through actin polymerization, where t-SNAREs and other fusion proteins have been found to be docked [57,58].

The fusion process to form multinucleated cells of monocytic origin has not been well characterized, although many attempts have been made to elucidate its mechanism. It has been proposed that fusion occurs in three programmed steps: first the cells acquire the ability to fuse, then the fusion-competent cells migrate and attach by their approximating membranes, and lastly the cells must fuse, sharing their cellular components and becoming a single entity [59]. The second step of fusion involves the cytoskeletal components that enable cell spreading and motility [60]. In FBGC formation, the presence of pseudopodia/filopodia as well as the sinking of the plasma membranes of one cell into another has been shown to occur during fusion [61,62]. Filopodia, which are composed of filamentous actin (F-actin) bundles, have been associated with processes such as chemotactic sensoring of the environment, controlling the direction of cell migration, and substrate adhesion in the formation of MGCs [63,64,65].

Binding of M-CSF to monocytes activates the DAP12/Syk signaling pathway, where DAP12 couples the activation through the cytoskeleton through the recruitment of Syk to create a cell that is fusion competent [66]. FBGC formation *in vitro* can be induced by monocytes through the introduction of IL-4 or IL-13 and subsequent cell fusion [67,68]. Cells created *in vitro* with these cytokines appear morphologically indistinguishable from those that are adherent to biomaterials or those in association with infectious granulomas. Fusion of macrophages in the formation of FBGCs requires adhesion success, which is highly dependent on the type of surface and absorbed blood proteins [69,70]. In cultures, unless there is a platform present that provides for adhesion of the monocytes, cytoplasmic spreading of the cells and fusion does not occur.

FBGCs that form on implanted surfaces are thought by many to degrade certain types of materials, obviously affecting their biocompatibility and their efficacy. Fusogens, molecules that facilitate the fusion of cell membranes, required for cell-cell fusion in the formation of FBGCs include DC-STAMP, E-cadherin, CD44, macrophage fusion receptor (MFR), and macrophage mannose receptor [71,72,73,74,75]. Generally, macrophages have a remarkable plasticity that allows them to change their phenotype based on environmental cues. However, FBGCs are terminally differentiated, but exhibit poor phagocytic abilities and have high levels of lysosomes [76]. It has been suggested that these cells cause chronic inflammation with osteolysis, subsequent failure of new bone formation, and also contribute to biomaterial degradation [77,78].

## 4. Wound Healing and Tissue Response

Inflammation is triggered by cells when they undergo injury and necrosis and is a vital step in the healing process [79]. There are four stages that occur in response to tissue injury; hemostasis, inflammation, proliferation, and remodeling [80]. Damage to the vasculature leads to activation of platelets by tissue factor in the injured tissues and subsequent clotting factors that initiate hemostasis. Provisional matrices consist of erythrocytes and fibrin and provide a platform for other cell types to migrate. Additionally, platelets release growth factors that recruit various cells types, including neutrophils, macrophages, and fibroblasts [81]. The first cells to arrive at the injury site are the neutrophils. While removing bacteria and debris, they also release additional chemotactic molecules to recruit macrophages [82]. The macrophages also secrete pro-inflammatory molecules, including platelet-derived growth factor, TNF α, IL-6, granulocyte-stimulating factor, and GM-CSF, in order to recruit more macrophages [83]. Classically activated macrophages appear to be the predominant cell type around 48 h after the initial injury [84]. The role of macrophages at this time point is to phagocytose debris and apoptotic cells, which can lead to conversion to a wound healing or reparative macrophage [81,85,86,87]. As mentioned above, T lymphocytes play a role in the polarization of macrophages to the wound healing phenotype through release of IL-4 and IL-13. The proliferative phase of wound healing involves an increase in cells, the formation of new blood vessels, and the deposition of extracellular matrix [81,88,89]. Reparative macrophages, along with fibroblasts, continue to resorb debris and begin to lay down new matrix, which along with factors released to promote angiogenesis, form granulation tissue [80]. The last phase is the remodeling phase which involves remodeling of the granulation tissue to form the mature tissue or scar through the work of matrix metalloproteinases and their respective tissue inhibitors [80]. Prolonged remodeling may occur in the presence of a foreign body and may lead to exuberant tissue fibrosis and scarring [80,90,91,92].

## 5. Protein and Cellular Response to Biomaterial Implantation

**Figure 3:**
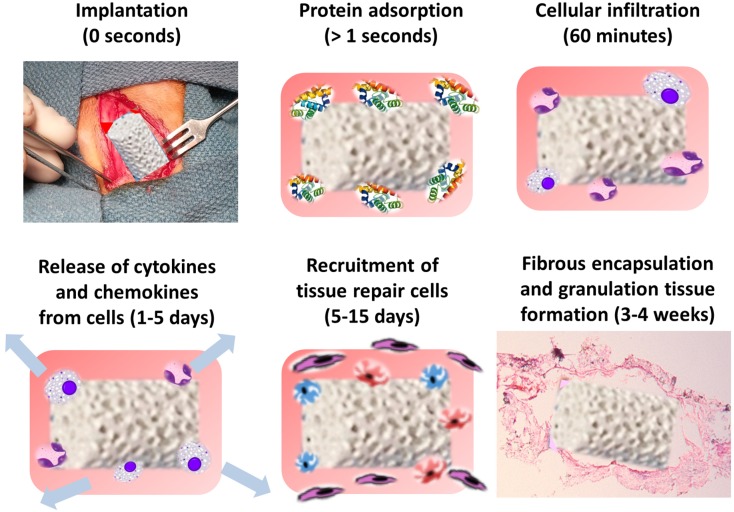
Immediately after implantation *in vivo*, a layer of proteins from the microenvironment adsorb and coat the surface of the biomaterial device. This protein adsorption results in attraction, infiltration, and attachment of various cell types such as monocytes, macrophages, and platelets. These cell types release chemokines and cytokines that recruit tissue repair cells to the site of inflammation. These cells produce collagen and result in the fibrous encapsulation of the implanted material.

Implanted devices and biomaterials immediately acquire a layer of host proteins (adsorption) prior to interacting with host cells (Figure 3). Proteins adsorbed onto the surface of biomaterials during the initial stages of hemostasis include albumin, fibrinogen, complement proteins, fibronectin, vitronectin, and globulins [93,94,95]. The types, levels, and surface conformations of the adsorbed proteins are dependent on biomaterial surface properties and are crucial determinants of the tissue reaction to such implants [13,96]. This ultimately dictates the adhesion and survival of cells, especially monocytes, macrophages, and FBGCs, on these protein-coated surfaces [14]. Macrophages respond rapidly to biomaterial implantation and are the dominant infiltrating cells [97]. They have been shown to respond and naturally bind to almost all biomaterials once implanted, including ceramics [98], cements [99], metal [100], polymers [101], and collagen [102,103]. In order to perform various functions, macrophages exhibit a wide range of transient polarization states that take cues from the microenvironment in which they are implanted.

As a morphologic variant, macrophages can fuse into MGCs, and become FBGCs, which are observed at the biomaterial--tissue interface of implanted devices and tissue engineering scaffolds [9,104]. It has been seen regularly that the process of bone formation is inhibited locally with the appearance of FBGC [105]. It has been demonstrated that macrophages may participate in the degradation of biomaterials and can contribute osteogenic and osteoinductive cytokines which aid bone regeneration and healing. The surface chemistry of the substrate onto which the MGCs adhere and the protein adsorption that occurs before cell adhesion may play crucial roles in the inflammatory and wound-healing responses to biomaterials and medical devices *in vivo* [97,106,107].

## 6. Macrophage Recognition and Attachment to Biomaterials

**Figure 4:**
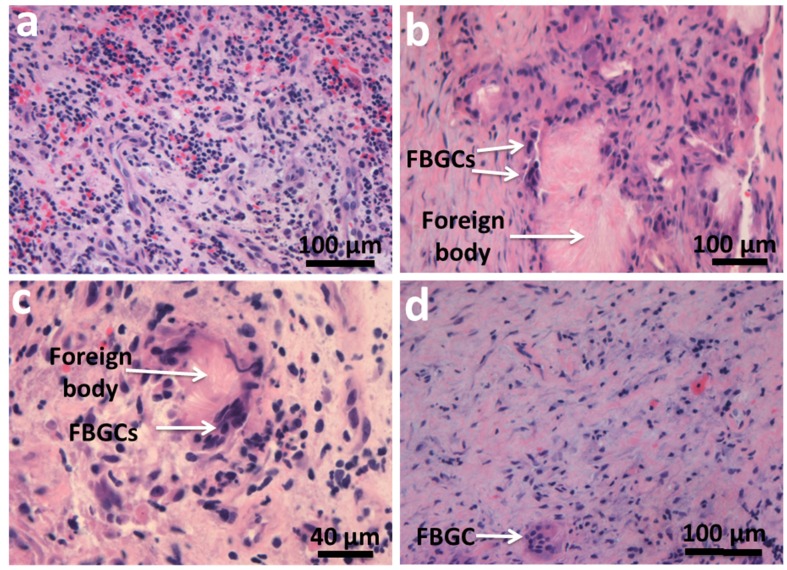
(**a**) Mixed acute and chronic inflammatory infiltrate; (**b**,**c**) FBGCs surrounding a foreign body; (**d**) Fibrous tissue and FBGC.

Synthetic artificial biomaterials present a novel challenge to macrophage recognition. However, macrophages react and respond to almost all the various biomaterials implanted [97]. Macrophages respond to foreign bodies due to their ability to recognize self and non-self. Antigen-presenting cells, which include macrophages and dendritic cells, are able to process antigens during their and present them to the cells of the adaptive immune system. Macrophage transmembrane proteins, including TLRs, scavenger receptors, and mannose receptors, all work to recognize specific ligands, from lipoproteins to bacterial DNA [108]. FBGCs are known to form in the presence of pathological conditions, one of which includes the presence of a foreign body, such as the placement of a biomaterial. Macrophage fusion to form a FBGC occurs through the induction of a response by tissue injury and the presence of foreign bodies or biomaterials (Figure 4). Physical features such as size, substrate stiffness, and topography, can elicit and determine the foreign body response in addition to surface chemistry, ligand presentation, degradation rates, and the release of growth factors. It has been well established that biomaterials produce micro-environmental cues that modulate the response of inflammatory cells [16]. Biomaterials are thought to activate the macrophage responses in a similar way to their reaction to Gram-negative bacteria or LPS, through the activation of TLR [109]. However, the adaptive immune system is not involved in the tissue response to biomaterials.

The response of tissues to a foreign body is much the same as the standard response to tissue injury, only with lengthened proliferation and remodeling phases. The initial stages of wound healing occur as they would in the response to wound healing; however, inflammation and macrophage activation do not resolve at the later stages and persistence of inflammatory cells, in particular macrophages, occurs. Macrophages use point-contacts and podosomes to undergo migration and perform mechano-sensing (haptokinesis) in order to interact with the adsorbed proteins using adhesion recognition proteins [110].

The mechanisms by which macrophages recognize different biomaterials are still not entirely known. It is thought that after implantation, proteins from the host (extracellular matrix and blood proteins) may be adsorbed on the surface of the biomaterials [111]. These proteins on the surface of materials can potentially trigger macrophage responses. A second proposed mechanism involves complement receptors on macrophages forming complexes with adsorbed complement proteins or IgG and IgM antibodies as part of the opsonization process [112]. Lastly, cell adhesion mediated by ligand–receptor complexes may be regulated by the presence of active cytokines and growth factors [111]. However, it has been observed that macrophages also respond to biomaterials *in vitro* where the above-mentioned factors are not present.

Macrophages bind to foreign materials through a mechanism involving integrin-driven interactions [13]. Integrins are transmembrane proteins that enable the interaction of cells with the extracellular matrix, through the combination of an alpha and a beta component. The binding of integrins to foreign materials is known to cause a change in cell movement, gene transcription, cell proliferation, survival, and alteration in the cellular cytoskeleton [113]. The expression profile of FBGCs includes the following integrins; α_M_β_2_, α_X_β_2_, α_5_β_1_, α_V_β_1_, α_3_β_1_, and α_2_β_1_ [114]. This indicates a strong role for subunits β_1_ and β_2_ as mediators of adhesion during FBGC formation. The proteins that have previously absorbed to the surface of the biomaterial, including fibrinogen, vitronectin, and some members of the complement pathway, are recognized via the β_2_-associated integrins [70,115]. Complement receptors on macrophages may complex with absorbed proteins as part of the complement cascade or IgG and IgM in order to activate the opsonization process [116]. As foreign materials are unable to be engulfed by a single macrophage, the cells undergo fusion to form FBGCs. As the size and multinucleation of the cells increase, the phagocytic abilities decrease, and the capacity for extracellular degradation progresses [102,117].

## 7. Macrophage-Mediated Phagocytosis of Biomaterials

**Figure 5:**
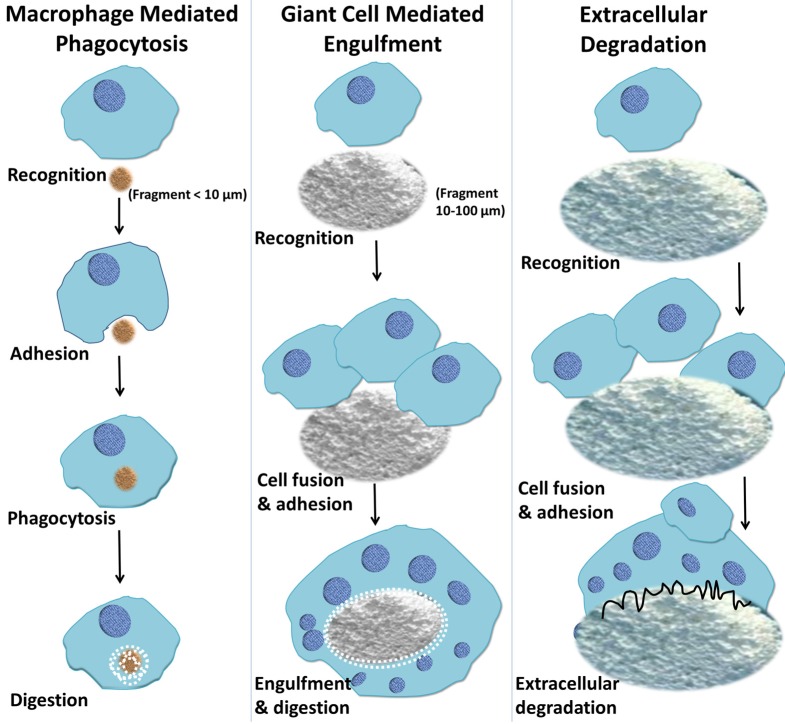
A schematic depiction of macrophage response to biomaterials depending on the size of the implanted materials. Macrophages respond to small fragments and particles (<10 μm in diameter) by internalization via phagocytosis and intracellular digestion. If the particle size is larger than 10 μm and smaller than 100 μm, the macrophages fuse together, forming giant cells which in turn engulf the particles and digest them. If the particles are larger, the bulk digestion is carried out via extracellular degradation by macrophages and macrophage-fused giant cells through the release of enzymes and/or pH lowering mechanisms.

Phagocytosis is the uptake of microbial bodies, particles and debris originating from implanted biomaterials [118]. The process of phagocytosis is initiated by the interaction of specialized plasma membrane receptors with specific molecular patterns of ligands, on the surface of a given particle [119,120]. Ligand–receptor binding initiates local reorganization of the actin-based cytoskeleton, which is responsible for the internalization of particles [121]. Macrophages respond to particulate biomaterials and engulf particles and fragments depending on their size (Figure 5) [122]. During phagocytosis, macrophage membrane reorganization leads to the complete envelopment of particles, which are contained within the cytoplasm in membrane-bound organelles known as phagosomes [123]. By a complex maturation process, lysosomes, hydrolytic enzymes and other substances are fused and released into phagosomes to kill, digest, and degrade the internalized particles [123]. When the particle sizes are beyond the capacity of a single macrophage to internalize (between 10 and 100 μm in diameter), FBGCs are formed (Figure 5) [97]. These cells then attempt to engulf large particles, succeeding at times and failing at others. However, they remain at the biomaterial–tissue interface for the lifetime of the implanted device [9].

## 8. Macrophage-Mediated Extracellular Degradation of Biomaterials

Macrophages, in addition to their function as phagocytes, are also involved in the extracellular biodegradation of biomaterial matrices such as collagen by the release of a variety of enzymes [124]. Macrophages and FBGCs release mediators of degradation such as reactive oxygen intermediates (ROIs), enzymes, and acid between the cell membrane and biomaterial surface [125,126]. It has been noted that phagolysosomes in macrophages can have acidity as low as pH 4 [127]. Biomaterial surfaces are susceptible to high concentrations of these degradative agents and the biomaterial surface chemistry dictates its susceptibility to biodegradation. Studies have focused on the ability of macrophage-derived enzymes to facilitate the degradation of polyurethanes [128,129,130,131,132,133]. Polyurethanes are condensation polymers that undergo degradation via the soft segment of the polyethers [134,135,136]. Adherent macrophages and FBGCs in the foreign body reaction are known to result in the degradation of polyurethanes by stress cracking on the biomaterial surface leading, ultimately, to device failure [14,137,138]. Polymers such as polyethylene, which is used in artificial joints, or polypropylene, which is used as a suture material, may undergo surface oxidation by ROIs [14]. However, devices and prostheses composed of addition polymers for *in vivo* implantation usually contain small amounts of antioxidants to inhibit this oxidative process. Studies have clearly shown that the use of antioxidants in polymeric biomaterial devices inhibits the oxidation process that occurs with the foreign body reaction [139,140]. 

Resorbable sutures are polyesters that are specifically designed to undergo degradation and complete resorption into monomer units that can be degraded in the Krebs’ cycle. Examples of these include polylactic acid, polyglycolic acid, polycaprolactone, and others. Degradation of some polyester biomaterials has also been associated with enzymatic degradation [141]. Although not all polyesters undergo degradation, polyethylene terephthalate (Dacron^®^, Vascutek Terumo, Inchinnan, Scotland) which is used as a vascular graft prostheses material, has demonstrated biodegradation *in vivo* [14]. Esterases secreted by monocyte*/*macrophages mediate polycarbonate-urethane biodegradation [101]. Although it has also been shown that phagocytosis is reduced after macrophage fusion, the extracellular degradation capacity of fused FBGC is greatly improved and can degrade polymers, collagen*/*hydroxyapatite composites and calcium phosphate cement substrates both *in vivo* and *in vitro* [97]. FBGCs have been shown to biodegrade polymeric devices and the rate of degradation is markedly increased underneath the cells [142].

Various bioceramics such as calcium phosphates are commonly used for bone regeneration and repair application [143,144,145], and are resorbed *in vivo* via passive dissolution and cellular processes [98,146,147,148]. Cell-mediated calcium phosphate resorption occurs due to the particle formation and fragmentation due to implant disintegration. The cells that take part in cell-mediated resorption are osteoclasts, which create an acidic environment on mineral surfaces by the release of protons, macrophages and FBGCs [14]. Monocytes/macrophages are among the first cells to colonize the surface of these calcium phosphate particles and play a crucial role in biodegradation [149]. Macrophages that encounter these particles are activated to endocytose, and resorption efficiency is inversely related to particle size [150]. Phagocytosis by monocytes/macrophages or acidic destruction by osteoclasts result in bio-resorption of calcium phosphate cements *in vivo* [151].

## 9. The Effect of Chemistry and Physical Features of Biomaterial Surface on the Foreign Body Response

The surface chemistry [152], form, and topography [153] of biomaterial surfaces determine the composition and severity of the foreign body reaction. With biocompatible materials, the foreign body reaction may be modulated by the surface chemical and physical properties [154], and by the relationship between the surface area of the biomaterial and the volume of the implant. High surface-to-volume implants such as porous materials show higher ratios of macrophages and foreign body giant cells than do the smooth-surface implants [155]. Implant sites that have a greater number of macrophages and foreign body giant cells have more fibrosis fibrosis and encapsulation of the biomaterials [155]. Research over the past few decades has been focused on modifying surface characteristics by various techniques including physical modifications, chemical modifications, and radiation [156,157]. *In vitro* studies have shown that the modification of biomaterial surface properties (including chemistry, domain composition, wettability, and morphology) has been shown to affect protein adsorption and subsequent cellular responses to biomaterials [152]. However, due to the use of surfaces that differ only in one or two properties and the lack of well-characterized animal implantation models, research studies have provided little understanding into the influence of surface properties on the pathogenesis of the foreign body reaction.

Biomaterial surface chemistry is known to play a role in the complement cascades that result in the recruitment and activation of phagocytes and adherence and activation of leukocytes [158], as observed in the initial work done on hemodialysis membranes [159]. It has been observed that the species, composition and density of the surface functional groups play an important role of controlling protein, cell and tissue reactions to implanted biomaterials [152]. It has been observed that biomaterials with altered chemical structures, by containing polyethylene oxide, have been shown to reduce protein adsorption [160,161,162], macrophage attachment [163], and experimental postoperative adhesions [164]. However, there is a need for more in-depth *in vivo* studies to define the interactions between surface functionality and host responses. Implantable biomaterials are predominantly hydrophobic and have high affinity to a several proteins [24,152]. As explained previously, immediately after implantation, the biomaterial surface is covered with a layer of plasma proteins [165,166], and these proteins, possibly via hydrophobic interactions, adopt an altered conformation and to expose the hydrophobic domains which are highly adherent to hydrophobic biomaterial surfaces [167,168]. The conformational changes observed in the adsorbed proteins are accountable for instigating adverse reactions such as inflammation, coagulation, and foreign body response [169,170,171].

Surface geometry has also been researched and explored with regards to understanding its influence on macrophage behavior and host responses. Substrates with micro-pattern grooves and ridges have been used to understand better macrophage behavior and foreign body reactions toward micro-sized topography [153,172,173]. Also, various studies have investigated macrophage and/or tissue responses toward nano-sized surface geometry [174,175,176,177]. We have already discussed the effect of the size of particles and degradation products of biomaterials. Experimental studies have also confirmed the role of particle shape in drug delivery via implantable biomaterials [178]. It is known that the local geometry of the particle at the point of cell attachment, not the overall particle shape, dictates whether macrophages initiate internalization [178]. It has been seen that elliptical-shaped discs (sufficiently small to be phagocytized), at the pointed end, are fully internalized by macrophages in a few minutes, whereas macrophages attached to the flat region of elliptical discs do not succeed for over 12 h [178]. The internalization of cylindrical particles depends strongly on their aspect ratio, with particles possessing an aspect ratio of three internalized about four times as fast as their spherical counterparts of the same volume [179]. It has also been seen that elongated worm-shaped polymeric particles show negligible phagocytosis compared with spheres of the same volume [180].

The nano- and microstructure of biomaterial surfaces plays a decisive role and affects cell morphology, adhesion and/or motility [181]. Also, fibrous capsule formation on a porous surfaces are often found to be thinner than that on dense solid implant surfaces [160,182]. Research has shown pronounced differences in cell spreading and focal adhesion dynamics, dependent not only on the feature size (50 and 500 nm), but also on the spacing between cell-recognizable features [183,184]. The influence of fiber diameter on fibrous capsule formation, a typical response of the body to isolate foreign bodies was studied by Sanders *et al.* [185]. It was observed that fibers having diameters ~6 μm or more supported fibrous capsule formation, whereas smaller diameter fibers did not. Interestingly, this trend was not affected by fiber material choice but was more dependent on the inter-fiber spacing [185]. This suggests that the local geometry might be more important in this context than the material chemistry.

Metals in contact with biologic systems can undergo corrosion [186,187] and release ions, which can activate the immune system by forming complexes with native proteins [188,189]. These metal-protein complexes can act as antigens for eliciting hypersensitivity responses [190]. In general, titanium and titanium alloys are considered to be biocompatible materials for implantation due to a layer of titanium dioxide (TiO_2_) that forms on the implant surface [191]. Released ions from titanium debris after implantation also have the potential to combine with native proteins forming a protein-metal complex, become immunogenic, and elicit a Type-IV (T-cell mediated) response [192,193,194,195]. Foreign body responses to implant surfaces have been identified in several experimental studies of osseointegration [196]; however, inflammatory responses to endosseous dental titanium implants are limited [197]. The success of dental implants *in vivo* is dependent on the material properties of the implant material including mechanical properties, surface chemistry, and surface topography [198]. The main goal after implantation is to obtain an appropriate host tissue response for the particular application [199,200,201] and surface topography is one of the chief determinants of implant performance by influencing cell behavior [198,202]. We know that macrophages are one of the first cells to arrive at the tissue–dental implant interface [203,204] and their interaction is thought to involve adhesion, activation, and secretion of cytokines at the implantation site [205,206]. Macrophages are known to prefer rough surfaces to smooth ones [207,208] and elongate significantly on grooved implant substrates [209]. Pro-inflammatory cytokines, such as IL-1and IL-6, and chemokines such as monocyte chemo-attractant protein (MCP)-1 and macrophage inflammatory protein (MIP)-1, along with tumor necrosis factor (TNF), are widely expressed at the early stages of implantation, and their presence directs host responses such as cellular recruitment and possibly bone formation [210]. It has been seen that the increase in surface roughness decreases osteoblast proliferation, increases differentiation, and increases protein synthesis and matrix production [211]. The increase in the surface area of a roughened surface has also been found to affect cytokine production, with macrophages secreting more IL-1 when exposed to titanium particles at higher surface area ratios [212]. Also, it is speculated that the rough surfaces may absorb more fibronectin than other surfaces [213] and, consequently, exhibit increased macrophage attachment [210].

The presence of macrophages around implants was initially thought to be detrimental to the osseointegration process [207,208]. Recent studies have indicated that macrophages play an important role and potentially might have a beneficial effect [100,199,214]. One study has showed that titanium surface topography modulates osteoinductive (BMP-2) and osteogenic cytokine (TGF-1) expression in macrophage cell lines [100]. Although neutrophils are seen to be recruited on the basis of pure wound healing phenomena, macrophages are only recruited if a biomaterial substrate is present [215]. Macrophages can further fuse into foreign body giant cells as explained in previous sections and these cells are found frequently on the dental implant surface [216]. This reinforces the concept that osseointegration is a foreign body reaction [217,218] because dental implants are foreign bodies themselves. Hence, osseointegration is the direct result of a foreign body reaction which, with the right intensity in the inflammatory response, balances itself out and allows for bone to ultimately grow on the implant surface ultimately [219]. Just like soft tissue implants, which end up encapsulated in poorly vascularized fibrous tissue, titanium dental implants also become surrounded and enclosed by condensed bone that is poorly enervated and vascularized. This is a typical result of a foreign body reaction that has reached equilibrium [217].

It is clearly evident that the chemistry and physical properties, such as size, shape and surface texture profoundly impact the function of an implanted biomaterial. Further work remains to map the dependence of biological response to physical properties and to categorize the relative effect and role of different physical and chemical factors towards foreign body reactions. For each application, comprehensive mechanisms of how physical properties affect biological performance and the interplay between various physico-chemical properties are required to be elucidated.

## 10. Conclusions

The development of new biomaterials for biomedical devices and tissue-engineered constructs requires an in-depth understanding of the biological responses to implanted materials. Once a biomaterial is implanted, a succession of events takes place leading to the formation of FBGCs at the biomaterial-tissue interface. However, the type of cellular and tissue response to biomaterials is dependent on the nature of the implanted biomaterials. Bulk materials such as implantable biomedical devices, pins, screws, plates, sutures, or membranes require the formation of FBGCs and the attachment of these cells to the surface of the biomaterials. These FBGCs may reside on the surface of the materials for the implant’s entire lifetime. It has been shown that macrophage responses to particulate biomaterials are dependent on the average particle size of the materials. Biomaterials with particle sizes smaller than a single-nucleated macrophage (~10 μm in diameter) are readily engulfed by macrophages via phagocytosis. Larger particles (between 10 μm and several hundred micrometers in diameter) are beyond the phagocytic capabilities of macrophages and, as a result, may be taken up within MGCs or FBGCs. The macrophage response to biomaterials is also dependent on the nature of the materials. Degradable biomaterials are degraded within phagosomes after phagocytosis, or eroded via extracellular resorption, with or without the involvement of FBGCs. Any associated inflammation is resolved after total resorption of the biodegradable materials has taken place. Non-degradable biomaterials do not degrade either within the macrophage phagosome, or by extracellular resorption. However, macrophages continue to infiltrate continuously, in an effort to phagocytose undigested particles or to fuse into FBGCs and remain on the surface of the implanted biomaterials. Hence, for biomaterials that do not degrade, it is important to choose those that evoke less macrophage response. Surface chemistry can have an effect on macrophage behaviors such as adhesion, fusion, apoptosis, and cytokine secretion. Macrophage responses to, and interactions with, biomaterials are currently not entirely understood. Further research to understand the mechanisms of macrophage mediated biomaterial degradation is required, and this is crucial for the improvement of biomaterials employed in tissue engineering applications. In order for the material-cell hybrid constructs to perform optimally, specific modulation of the foreign body reaction is required. The biomaterial should provide a biomimetic environment to ensure cell survival and also direct cell migration to ensure that relevant cells migrate to and adhere to the implant. 

Interest in understanding macrophage-biomaterial interactions exists on two distinct levels. Understanding the particular aspects of the cell-surface interaction that initiate adhesion and activation of macrophages can then lead to the designing of novel biomaterials that encourage favorable macrophage-biomaterial surface interactions for clinical applications. It is now well known that the physical properties such as topography, stiffness, porosity, pore-size, chemical properties and degradation rates of the implanted biomaterials all influence cell behavior. The effect of such physico-chemical cues on immune cells, specifically macrophages, is not well understood. This lack of understanding of macrophage responses is compounded by the complexity of interplay present between biomaterial properties and those that result from interactions with the local micro-environment as a consequence of biomaterial/tissue interaction. While several cell types are involved in tissue healing post injury, macrophages play a vital role in mediating tissue remodeling and regeneration by secreting cytokines and chemokines that directly impact the tissue repair processes. Therefore, elucidation of the exact role of macrophages in tissue healing processes, especially in events that follow biomaterial implantation, will aid in the design of novel biomaterials and tissue-engineered constructs that elicit a favorable immune response upon implantation and perform suitably in their intended applications.

Currently, implantable sensors utilizing nanotechnology are at the forefront of diagnostic and medical monitoring technologies. These sensors often include nano-structured carbon allotropes, such as graphene or carbon nanotubes, because of their unique and enhanced properties [220]. The age of carbon nano-materials is just beginning and is expected to have a major impact in many areas along with nano-fibers that have enormous potential as wound dressings and other clinical applications [221,222,223]. It is required in the future for *in vivo* studies to be conducted that investigate the interactions of these implantable sensors fabricated with the cellular components of the immune system. This will provide crucial cues as to how the body tissues react to these materials and will help fine-tune and develop better technologies that are immune-compatible.
